# Retraction Note: Long noncoding RNA neuroblastoma-associated transcript 1 gene inhibits malignant cellular phenotypes of bladder cancer through miR-21/SOCS6 axis

**DOI:** 10.1038/s41419-022-05485-2

**Published:** 2022-12-07

**Authors:** Zhongyuan Liu, Dalong Xie, Hui Zhang

**Affiliations:** 1 Department of Urinary surgery, Shengjing Hospital, China Medical University, Shenyang, 110004 China; 2 Department of Anatomy, College of Basic Medical Science, China Medical University, Shenyang, 110122 China

Retraction Note to: *Cell Death and Disease* 10.1038/s41419-018-1090-z, published online 11 October 2018

The editors have retracted this article. Concerns were raised regarding a number of figures in this article, specifically:

- The bottom right panel of Fig. 1 appears to overlap with the bottom right panel of Fig. 3F
- The bottom left panel of Fig. 6 appears to overlap with the bottom right panel of Fig. 7D
- The bottom middle panel of Fig. 4D appears to overlap with the bottom middle panel of Fig. 7

The Editors therefore no longer have confidence in the reliability of the data reported in the article. Zhongyuan Liu and Dalong Xie have not responded to any correspondence form the publisher about this retraction; the publisher was unable to contact Hui Zhang.

